# Increased antinuclear (anti-Sm) antibodies in infertile patients with peritoneal endometriosis

**DOI:** 10.5935/1518-0557.20240070

**Published:** 2025

**Authors:** Renata Fogaça Borges, Vanessa Krebs Genro, Carlos Augusto Bastos de Souza, Tatiana Ferreira Michelon, Isabel Meneghetti Coimbra, João Sabino Lahorgue da Cunha-Filho

**Affiliations:** 1 Universidade Federal do Rio Grande do Sul, Porto Alegre, Brazil; 2 Hospital de Clínicas de Porto Alegre, Universidade Federal do Rio Grande do Sul, Porto Alegre, Brazil; 3 Department of Obstetrics and Gynecology, Universidade Federal do Rio Grande do Sul, Porto Alegre, Brazil

**Keywords:** endometriosis, infertile, anti-Sm autoantibodies, anti-RNP autoantibodies, anti-SS-A antibodies

## Abstract

**Objective:**

The aim of this study is to evaluate the intensity of five antibodies against extractable nuclear antigens (ENA: RNP, ScL-70, SS-B, SS-A, and Sm) in infertile patients with endometriosis.

**Methods:**

We investigated infertile women with minimal/mild endometriosis (n=43) and fertile women (n=46).

**Results:**

The intensity of immunoreactions was also similar for anti-RNP and anti-SS-A antibodies. However, the intensity of immunoreactivity for anti-Sm antibodies was higher in the endometriosis group.

**Conclusions:**

Patients with minimal/mild endometriosis have higher levels of anti-Sm autoantibodies.

## INTRODUCTION

Endometriosis is a prevalent disease that is typically associated with chronic pelvic pain or infertility. The precise mechanism for establishing and developing this disease has yet to be fully established. However, several authors have defined an essential and irrefutable role for the immune system in the pathophysiology of endometriosis (Herington *et al.*, 2011).

Antinuclear antibodies (RNP, ScL-70, SS-B, SS-A, and Sm) represent a heterogeneous group of antibodies that are associated with several autoimmune manifestations and immunological diseases, such as Lupus, systemic sclerosis, polymyositis, arthritis, and Sjögren’s syndrome (Alspaugh *et al.*, 1976; Sharp *et al.*, 1976; Moroi *et al.*, 1980).

Moreover, various reports have attempted to connect endometriosis to several autoimmune conditions (e.g., asthma, Lupus, allergies, hypothyroidism, chronic fatigue syndrome, rheumatoid arthritis, and fibromyalgia) (Alspaugh *et al.*, 1976; Sharp *et al.*, 1976), and some reports have identified associations between endometriosis and autoimmune manifestations (Sharp *et al.*, 1976; Moroi *et al.*, 1980; Herington *et al.*, 2011; Borrelli, 2015).

A well-designed cohort study evaluated the co-occurrence of endometriosis and some autoimmune manifestations (associated with multiple sclerosis, Lupus, and Sjögren’s syndrome), and these authors described a slight association between unselected endometriosis cases and autoimmune manifestations (Nielsen *et al.*, 2011).

Moreover, some autoimmune diseases like Lupus and antiplatelet antibodies are related to endometriosis (FerrariSouza *et al.*, 2023; Loyau *et al.*, 2023). The link between endometriosis and the immune system is extensively studied to find a cause-effect relationship to explain the development of this disease and the different phenotypes (superficial, deep, ovarian). This link (endometriosis and immune system) is also fundamental to investigating and developing a new therapeutic option, targeting the immunomodulation of those endometriosis patients (Yang *et al.*, 2023).

Therefore, considering that the precise intensity of the autoantibodies detected in cases of endometriosis has not been well studied or adequately described, the present study was designed to evaluate the mean intensity of five different antibodies against the extractable nuclear antigen (ENA: RNP, ScL-70, SS-B, SS-A, and Sm) in infertile patients with endometriosis.

## MATERIAL AND METHODS

A prospective case-control study included 43 patients with endometriosis and 46 healthy controls. Cases were defined as patients who underwent laparoscopy for endometriosis or infertility investigation whose findings were proven (laparoscopy and biopsy) endometriosis according to the ASRM classification. Controls were fertile patients who underwent laparoscopy for tubal ligation (contraception) without peritoneal endometriosis. All subjects were recruited from the ambulatory of endometriosis at the Hospital de Clínicas de Porto Alegre between 2016-2021. Exclusion criteria for both groups were evidence or suspicion of any autoimmune disease or clinical manifestations related to these diseases, other findings in the laparoscopy such as cysts or malignancies, and partners with male factor were also not included in this study. Patients were all negative for anti-thyroid antibodies, had normal serum PRL and TSH levels, and had regular menses.

A serum sample was collected at the time of anesthesia induction. A multiplex semi-quantitative immunofluorescence assay was performed to analyze five ENA-autoantibodies (Sm, RNP, Scl-70, SS-A, SS-B) in the same reaction using a commercial kit (Quantaplex INOVA Diagnostics Inc., San Diego, CA) with a Luminex flow cytometer. The prevalence of autoantibodies and their intensity were compared between both groups. The mean fluorescence intensity (MIF) was calculated for each antibody according to the manufacturer’s instructions. According to pre-established guidelines developed for Lupus patients, MIFs greater than 20 UL were considered positive. Fisher’s exact test or the chi-square and Student’s t or Mann-Whitney tests were applied, with significant results demonstrating *p* values <0.05 (IC95%). Multivariable analysis was done using linear or binary regression analysis.

The project was approved by the Hospital de Clínicas de Porto Alegre ethics committee, where the research was conducted (IRB equivalent).

## RESULTS

The baseline characteristics of patients included in this paper are the following: The mean age was similar between groups (cases 32.0±4.4 *vs*. controls 33.0±5.5; *p*=0.374). Endometriosis patients had lower BMI values than controls (23.7±4.3 *vs*. 26.3±4.1, *p*=0.006). However, the BMI values were not associated with the presence of specific antibodies in the overall sample (*p*>0.05) or among patients with endometriosis (*p*>0.05).

The overall prevalence of anti-ENA antibodies in this sample was 19.1% (17/89), similar in the cases and controls (20.9% x 17.4%; *p*=0.877). No cases were demonstrating positive anti-Scl-70 or anti-SS-B antibodies in the study group. Anti-RNP antibodies were similarly detected in 2.2% of cases and 2.3% of controls (*p*=1.000); anti-SS-A antibodies were detected in 14.6% of the sample (14.0% cases x 15.2% controls; *p*=1.000); and anti-Sm antibodies were detected in 5.6% of the sample (9.3% x 2.2%, respectively; *p*=0.193).

Moreover, the MIF values were similar between cases and controls for anti-RNP and anti-SS-A antibodies (RNP: 7.7±6.2 x 6.7±2.8, *p*=0.316; SS-A: 21.9±56.5 x 24.6±71.4, *p*=0.522, respectively). In addition, the prevalence of anti-ENA antibodies among patients with minimal endometriosis (GI) was 27.5% (8/29), whereas that among patients with mild endometriosis (GII) was 7.1% (1/14) (*p*>0.05).

However, the anti-Sm MIF values were higher in the endometriosis group (16.0±10.8 x 11.7±3.4, *p*=0.007).

## DISCUSSION

The current study was the first to describe an association between peritoneal endometriosis and anti-Sm autoantibodies. The originality and value of this report is the inclusion of a homogeneous group of endometriosis patients (only peritoneal, minimal/mild stage patients), as the central problem of most papers regarding endometriosis and the immune system involves the inclusion criteria applied and, consequently, the comparison between the experimental and control groups.

Our report states that endometriosis is linked to some autoimmune diseases, primarily Lupus (Nielsen *et al.*, 2011). Moreover, the Sm (Smith) antigen is a saline-soluble, non-histone glycoprotein, which is not dependent on DNA or RNA for its antigenicity. Antibodies to the Sm antigen are considered to be a specific serologic marker due to their high degree of specificity for systemic Lupus (SLE). In addition, these antibodies are observed in up to 30% of SLE patients and have been associated with the activity of this disease.

The most robust and convincing study connecting endometriosis to certain connective diseases described a population-based finding with a modestly (less than 60%) increased risk of Lupus among endometriotic patients (Nielsen *et al.*, 2011). Our findings are in agreement with these authors and demonstrated that although auto-Sm antibodies were higher in patients with endometriosis, this increase was limited and observed in less than 10% of the studied subjects.

Recently, our group also confirmed this finding, demonstrating a link between endometriosis and Lupus (Ferrari-Souza *et al.*, 2023).

Anti-DNA antibodies are a group of nonspecific antibodies that are associated with several connective diseases and manifestations. Furthermore, the presence of these antibodies may be linked to some of the clinical and laboratory findings of deterioration in these diseases (Kavanaugh & Solomon, 2002). The link between the immune system and endometriosis is unequivocal; however, most published papers have only explored associations with particular immune pathways, cytokines, or cellular functions (Eisenberg *et al.*, 2012). Furthermore, the main flaw of these previous studies is their need for a physiological explanation or the comprehension of background factors that link the findings to endometriosis.

In the current study, we found that infertile patients with minimal/mild endometriosis generally had higher levels of Sm autoantibodies. These complexes can likely disturb connective (collagen IV) tissue and vascular compartments (laminin and heparin sulfate) (Hahn, 1998), thereby promoting cellular invasion and neoangiogenesis in this group of patients. The immunological pathway related to anti-DNA antibodies involves T Helper cells and, in some cases, is also B cell dependent. In addition, specific cytokines and HLA class II molecules may also be involved (Hahn, 1998).

Autoimmunity seems to be associated with endometriosis, as several papers have documented this phenomenon (Lucena & Cubillos, 1999; Maeda *et al.*, 2002; Ulcova-Gallova, 2005). However, the study and control groups of all papers differ and are highly heterogeneous. Moreover, the prevalence of anti-DNA antibodies should not be the main factor for comparing our findings to those of other studies; instead, it is most important to describe a rational and well-defined immunologic pathway and to study why specific dysfunctions are related to the characteristics and grade of endometriosis.

Furthermore, certain autoimmune conditions, such as Lupus and antiplatelet antibodies, are connected to endometriosis (Ferrari-Souza *et al.*, 2023; Loyau *et al.*, 2023). The relationship between endometriosis and the immune system is extensively researched to elucidate a cause-andeffect connection, explaining the disease’s development and its various phenotypes (superficial, deep, ovarian). This connection is pivotal for exploring and creating therapeutic approaches that target immunomodulation in individuals with endometriosis.

Our results are in agreement with the idea that mild/ minimal endometriosis is an active immunological disease, even more so than moderate or severe endometriosis, which could explain why treatments such as immune modulation and IVF protocols could increase pregnancy rates in these patients (Ulcova-Gallova, 2005).

However, our research has several limitations. First, we included only minimal/mild endometriosis, preventing our results from being extrapolated to all endometriosis stages and sub-types. Second, it was impossible to perform a temporal evaluation or connection between endometriosis and the positivity of these autoantibodies. Consequently, our results indicate that only an association and a functional test are required to understand better and elucidate the precise immune mechanisms involved.

## CONCLUSION

In conclusion, we demonstrated for the first time that the prevalence of anti-ENA antibodies among patients with endometriosis GI/II is similar to that of the healthy female population (19%). However, anti-Sm antibodies were modestly elevated in the studied infertile patients with peritoneal minimal/mild endometriosis. Thus, this study introduces a new perspective in terms of understanding the intensity of anti-Sm antibodies in endometriosis (understanding the Th2/T Helper and NK interaction) and the utility of these antibodies for treatment purposes.
